# Evolutionary overview of sarcopenia – bibliometric study on the Web of science: A review

**DOI:** 10.1097/MD.0000000000034500

**Published:** 2023-07-28

**Authors:** Juan Chen, De-jun Wang, Yi-chen Zhang, Yan-hong SU

**Affiliations:** a Key Laboratory of Sports Human Body Science, College of Physical Education, Liaoning Normal University, Dalian, China; b Hubei Daye Sixth Experimental School, Daye, China.

**Keywords:** sarcopenia, SciMAT, strategic coordinate analysis, theme evolution

## Abstract

Sarcopenia is an age-related degenerative disease associated with adverse outcomes such as falls, functional decline, weakness, and mortality. Exploring the dynamic evolutionary path and patterns of sarcopenia research topics within a temporal framework from the perspective of strategic coordinate maps and data flow can help identify the development rules of sarcopenia themes. After searching, a total of 16,326 articles were obtained. There are few early research topics, but the development maturity of the topics is high; the number of late research topics continues to increase, showing a trend of diversified development. The differentiation and fusion of the theme evolution path are obvious, and the theme inheritance index is high. The development trend of this research field is promising. The mature and stable professional topics such as “RESISTANCE EXERCISE” and “SURVIVAL” that appeared in the late stage belong to the core topics, while newly emerging topics like “FRACTURES” and “PROTEIN” belong to the marginal topics, indicating that the research on muscle and bone metabolism in the field of sarcopenia has yet to be further in-depth, and the “CANCER” topic is a highly promising research topic with strong development potential.

**Key Points**

**Aim:** The purpose is to study the dynamic evolution path of the field of sarcopenia research.
**Findings:** The development trend of this research field is good. The research on the direction of muscle and bone metabolism has yet to be further in-depth, and the topic of CANCER has great potential for development.
**Message:** This study shows that the hot spots and trends in future research on sarcopenia are reflected in related aspects such as muscle and bone metabolism, survival, and intervention.

## 1. Introduction

Population aging is an important trend in the demographic changes of many countries. The degree of population aging in various countries around the world is deepening, and the trend is irreversible. To actively address population aging, it is important to construct corresponding policy systems and social environment, accelerate the development process of aging careers and industry, and strengthen health guidance and comprehensive intervention for common and chronic diseases in the elderly, to improve the quality of life of the elderly, and reduce medical burden. Sarcopenia is one of the important diseases that affect the health and quality of life of the elderly. It is an age-related disease attributed to the aging process, which leads to the loss of muscle mass and function accompanying aging. This may lead to a decline in physical fitness and quality of life of the elderly, increase the risk of falls and disability, and incur high medical expenses.^[1,2]^

Sarcopenia is associated with adverse health outcomes and premature death, and has been included in the International Classification of Diseases with the code M62.84.^[3]^ The latest systematic evaluation study found that using different classifications and critical points, the prevalence of sarcopenia varied between 10% and 27% in the studies included in the meta-analysis.^[4]^ Sarcopenia has a relatively short research history, first proposed by Irwin Rosenberg in 1989. With the accelerated aging process, the field of sarcopenia has rapidly developed in the past decade. Tracking the research topics of sarcopenia and identifying its development patterns can help researchers better understand the development dynamics of sarcopenia and promote further development in the field.

Currently, sarcopenia research involves multiple aspects, including multi-group analysis,^[5]^ quality of life,^[6]^ drug therapy,^[7]^ biomarkers,^[8,9]^ related influencing factors,^[10,11]^ correlation with other diseases,^[12]^ exercise intervention,^[13,14]^ nutrition,^[15]^ molecular mechanisms,^[16]^ and measurement methods such as dual-energy X-ray absorptiometry for measuring muscle mass,^[17–19]^ bioelectricity impedance analysis and other measurement methods. Relevant studies conducted a descriptive and bibliometric analysis using Microsoft Excel, VOSviewer, and CiteSpace by retrieving articles and reviews with “sarcopenia” in their titles in the SCIE database from 1999 to 2021, and found that epidemiology of sarcopenia in different diagnostic tools and populations the recent research hotspot.^[20]^ In summary, the relevant research mainly focuses on the elaboration of the latest research results in the field of sarcopenia. Although a small number of studies involve the literature measurement analysis of interventions for sarcopenia, there have been no studies to discuss the changes in the research topics of sarcopenia in different periods and the fusion and differentiation paths of the topics. As a result, the historical development process of sarcopenia research topics and their future potential development directions are still unclear. Therefore, this article uses relevant literature in the field of sarcopenia as the research object and analyzes the research topics and dynamic evolution process of the field in different periods, providing a reference for relevant scholars to grasp the development trend, potential hotspots, and cutting-edge of sarcopenia.

## 2. Research methods

Bibliometrics is a measurable information method, commonly used to discover top journals and authors in a certain field, identify research progress, and predict research trends.^[21]^ SciMAT software (https://sci2s.ugr.es/scimat/), was developed by the University of Granada in Spain in 2012. By establishing a keyword co-occurrence matrix and clustering topics, it can present keyword evolution maps, strategic maps with density and centrality as the axis, and topic evolution maps drawn based on time processes, thereby revealing the dynamic evolution path of research topics over time. The software contains all processes and algorithms from data preprocessing to visualization of results. Compared with other bibliometrics visualization tools, SciMAT has unique advantages in expressing topic evolution and performs well in longitudinal time series analysis.^[22]^

**Figure 1.**
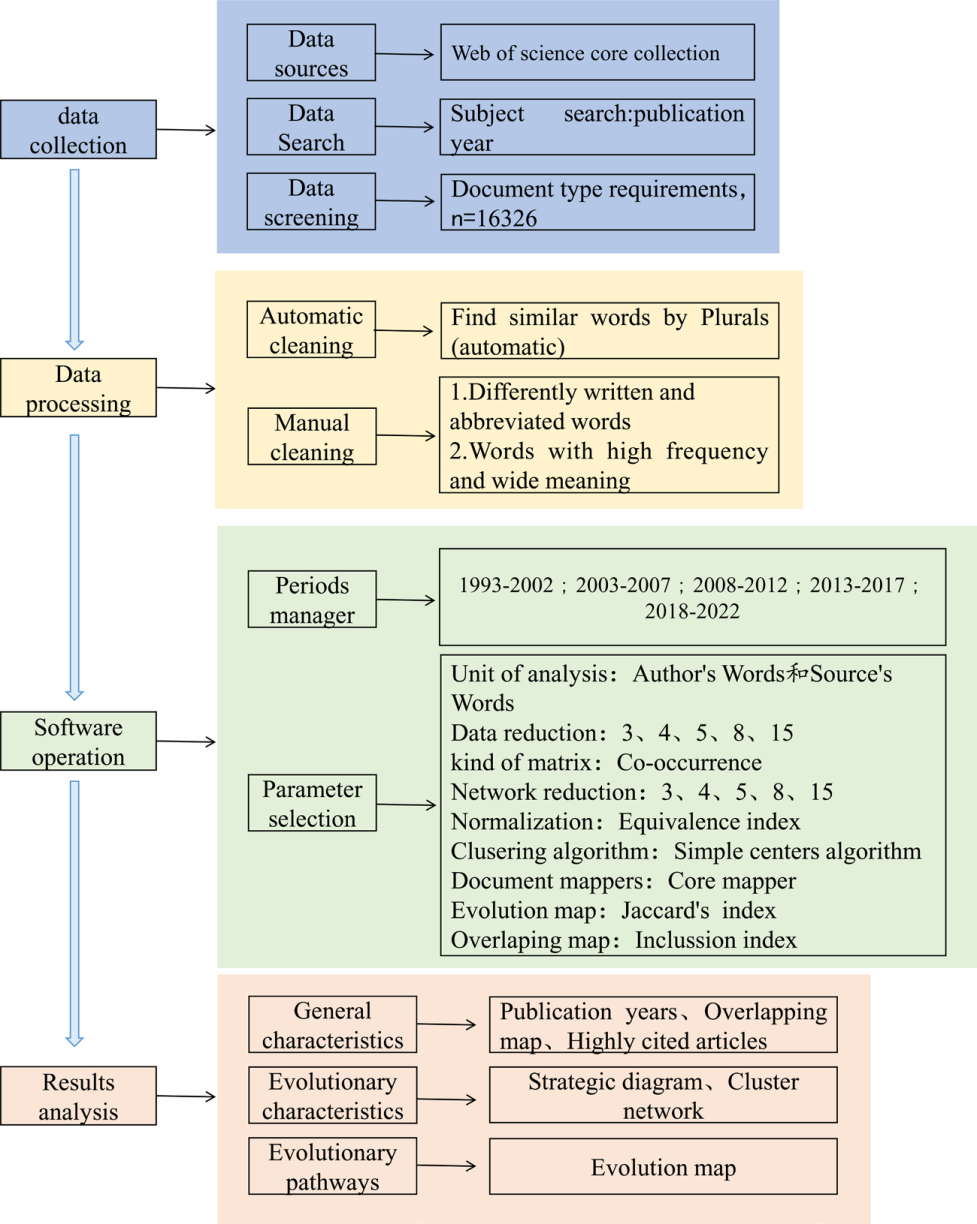
Framework for evolutionary analysis of research topics on sarcopenia.

This paper uses the SciMAT tool to draw a knowledge map of the research topic. Using the strategy chart and data flow, the research topics and their evolution in different research periods are analyzed, and the dynamic evolution path and evolution law of the research topics of sarcopenia are discussed. The analysis framework of this paper is shown in Figure 1, which is mainly divided into 4 parts: data collection, data processing, software operation, and result analysis.

## 3. Data acquisition and processing

### 3.1. Data sources and retrieval strategies

Search the core collection of the Web of Science. The search adopts a combination of keywords and free words. Find keywords (sarcopenia) and free words (sarcopenias) through the PubMed website. The search mode is Topic Search = (sarcopenia) or (sarcopenias). The search date is January 28, 2023. Select the document type (Article, Review Article), a total of 16,326 data were obtained, and the time was 1993 to 2022.

### 3.2. Data cleaning

In order to accurately obtain research topics in this field, it is necessary to perform data cleanup. Through the data cleaning function that comes with the SciMAT software, the singular and plural keywords are automatically merged, and the keywords that are not merged due to different writing and abbreviations are manually merged, such as “Functional capacity anti-oxidant” and “Functional capacity antioxidant,” “cut off value” and “cut off point”; finally, the frequency is higher but the meaning is broad and the directivity is weak, which may affect the connection between other keywords, such as “falls,” “expression,” “sample” and so on.

### 3.3. Study time zone division

According to the derived results, the research topic paper on sarcopenia was first published in 1993, so the research literature time in this article is 1993 to 2022. According to the research,^[23]^ the half-life of clinical medical journal papers is 4.515 years. This study takes 5 years as a cycle, but due to the small number of early publications in the field of sarcopenia, 1993 to 2002 is divided into the first time zone; the interval from 2003 to 2022 is divided into a 5-year cycle. According to this, the literature collection is divided into 5 time zones: 1993–2002, 2003–2007, 2008–2012, 2013–2017, and 2018–2022.

### 3.4. Parameter selection

The selection unit is analyzed as words (Author’s Words and Source’s Words), and the data reduction thresholds for the 5-time periods are 3, 4, 5, 13, and 15, respectively. The matrix type selects the co-occurrence matrix, and the network reduction thresholds are 3, 4, 5, 13, and 15. The standardized network similarity index selects the “Equivalence index,” the clustering algorithm selects the “Simple centers algorithm,” the graph display selects the “Core mapper,” the evolution graph similarity index selects “Jaccard’s index,” and the Overlap graph similarity index selects the “Inclusion index.”

## 4. Results

### 4.1. Statistics on the number of articles issued

**Figure 2.**
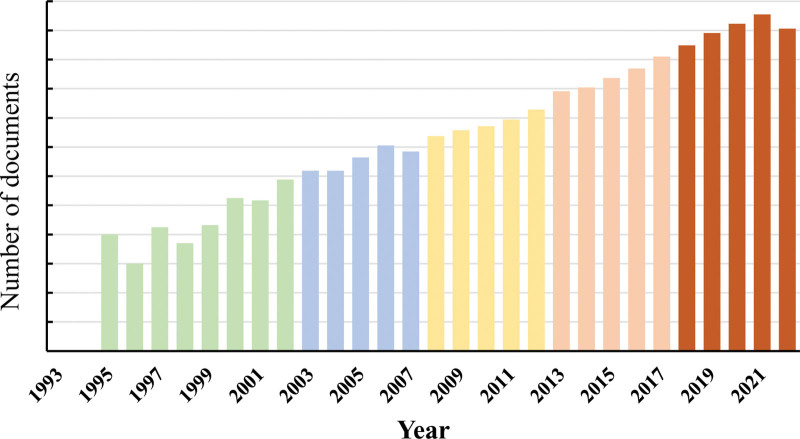
Annual publication statistics.

The number and trend of articles published in a certain field every year can reflect the attention of scholars to this field. Figure 2 shows a statistical analysis of the volume of papers published in the field of sarcopenia research from 1993 to 2020. The number of published papers in 1996 and 1998 decreased slightly compared with the previous year, showing an obvious upward trend on the whole.

The number of documents related to sarcopenia can be divided into 3 stages: slow growth, stable growth, and rapid growth. During the period from 1993 to 2007, there was a slow growth rate with average yearly publications of 47 papers. This indicates that sarcopenia began receiving attention during this period, leading to related theoretical and practical research. From 2008 to 2017, there was a stage of stable growth characterized by an average of 464.1 papers published each year. Since 2018, there has been a significant increase in the number of articles published, with 3010 articles published in 2021 alone. From 2008 to 2022, the volume of papers published in this field has grown steadily and rapidly, with an average of 2352.2 papers published every year. While the volume of papers published in 2022 is slightly lower than that of 2021, it is still indicative of the ongoing growth in the field of sarcopenia. The development of basic and clinical research in the field of sarcopenia has been effectively promoted by the consensus and guidelines for diagnosis and treatment formulated and issued by various sarcopenia working groups. This anticipates that the overall volume of publications in the field of sarcopenia will continue to show a rapid growth trend in the coming years.

### 4.2. Topic evolution coverage graph analysis

**Figure 3.**
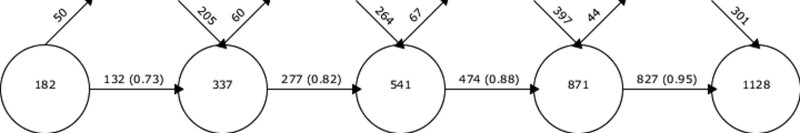
Coverage of research on the subject of sarcopenia during the research period from 1993 to 2022 (from left to right, they are the research period from 1993 to 2002, 2003 to 2007, 2008 to 2012, 2013 to 2017, and 2018 to 2022).

The theme evolution coverage map indicates the number of themes with keywords and presents the emergence, disappearance, and stability of research themes in the field in the form of data streams. As shown in the coverage map in Figure 3, the circle represents the study period, and the number inside the circle represents the number of themes in that period. The horizontal arrow between 2 circles indicates the continuity of themes in 2 research periods, and the number in the middle of the arrow is the number of shared themes between the 2 research periods. The number in the parentheses is the stability index, reflecting the continuity of the theme. The arrow pointing down from the upper left of the circle represents the number of new themes in that period, and the arrow pointing up from the upper right of the circle represents the number of themes that disappeared in that period.

New research topics continue to emerge in the field of sarcopenia, accompanied by an increasing stability index between research periods. Figure 3 shows the overlap of the number of themes in the field of sarcopenia research between 1993 and 2022. It clearly shows the emergence and disappearance of themes in the field, from left to right, for the research periods of 1993–2002, 2003–2007, 2008–2012, 2013–2017, and 2018–2022. The number of themes for each research period is 182, 337, 541, 871, and 1128, respectively. The number of themes retained in each period is more than the number of those that disappeared. The number of newly added themes and the total number of themes in each stage keeps increasing, indicating that the field of sarcopenia research is in a stage of vigorous development.

From 1993 to 2002, a total of 132 topics were carried over to the next research period, with a stability index of 0.73 for the next research period. During this research period, research in the field of sarcopenia was still in a slow-growing phase, belonging to the basic research stage. The number of new topics in the period from 2003 to 2007 was 205. In the research period from 2008 to 2012, 277 topics from the previous period were continued, with a stability index of 0.82 for 2 research periods. In the period from 2013 to 2017, there were 397 new topics, with 474 topics continuing from the previous research period. The stability index for the 2 research periods was 0.88, reflecting a stable growth in research in the field of sarcopenia and a stable increase in research articles. In the period from 2018 to 2022, there were 301 new topics and 827 topics continuing from the previous research period. The stability index for this research period and the previous research period was 0.95. With this, the number of topics and stability index has continued to increase, reflecting the gradual expansion of the research field of sarcopenia and the increasing richness of research content.

### 4.3. Analysis of highly cited literature

In the top 10 highly cited articles in the field of sarcopenia research, 3 articles are from the 1993 to 2002 research period,^[24–26]^ 4 articles are from the 2008 to 2012 research period,^[27–30]^ 2 articles are from the 2013 to 2017 research period,^[31,32]^ and 1 article is from the 2018 to 2022 research period^[33]^ (Table 1).

**Table 1:** The top 10 highly cited articles in the research topics of sarcopenia in the research period from 1993 to 2022.

Source documents	Published journals	Year	Citations
Cruz-Jentoft et al	*Age and Aging*	2010	7185
Cruz-Jentoft et al	*Age and Aging*	2019	3927
Fearon et al	*Lancet Oncology*	2011	2884
Baumgartner et al	*American Journal of Epidemiology*	1998	2558
Chen et al	*Journal of the American Medical Directors Association*	2014	2295
Janssen et al	*Journal of the American Geriatrics Society*	2002	2009
Fielding et al	*Journal of the American Medical Directors Association*	2011	1867
Prado et al	*Lancet Oncology*	2008	1851
Janssen et al	*Journal of Applied Physiology*	2000	1630
Martin et al	*Journal of Clinical Oncology*	2013	1478

During the 1993 to 2002 research period, 3 articles ranked fourth, sixth, and ninth in terms of citation frequency. This reflects that the papers published during this period constituted the research foundation of the field of sarcopenia research and played a fundamental role in research in this area. Baumgartner et al^[24]^ developed a human metrological equation to predict skeletal muscle mass for assessing sarcopenia. Sarcopenia was defined as 2 standard deviations below the mean value of the reference group for 4 limb skeletal muscle mass (kg)/height squared (m^2^) and was used for large-scale screening. The study found that the prevalence of sarcopenia in the population below 70 years old was 13% to 24%, while it was 50% in those aged 80 years and older. Another frequently cited study, with 1971 citations, used bioelectrical impedance to measure skeletal muscle mass. The subjects with skeletal muscle mass index lower than one to two standard deviations below the mean value for young adults (18–39 years old) were considered to have grade I sarcopenia, and those with skeletal muscle index lower than 2 standard deviations were considered to have grade II sarcopenia.^[26]^ These 2 studies on the measurement and application of sarcopenia laid the foundation for subsequent research.

During the 2008 to 2012 research period, 4 articles ranked first, third, seventh, and eighth in terms of citation frequency. The most cited article was the 2010 European Working Group on Sarcopenia in Older People consensus definition and diagnosis of sarcopenia in older adults. The article defined sarcopenia as a syndrome characterized by progressive and generalized loss of skeletal muscle mass and strength, and recommended the use of low muscle mass and low muscle function to diagnose sarcopenia, as well as published various indicators measurement methods and cutoff points.^[28]^ In 2011, the International Sarcopenia Consensus Conference Working Group published a consensus statement, emphasizing that special consideration should be given to patients who are bedridden, unable to stand up independently from a chair, or have a gait speed of less than 1 m/s. Patients who meet these criteria should undergo further body composition assessment using dual-energy X-ray absorptiometry.^[30]^

During the 2013 to 2017 research period (Table 1), Martin et al^[31]^ studied the correlation between muscle depletion and prognosis in cancer patients and found that rapid weight loss, low muscle index, and low muscle attenuation were independent prognostic factors for survival. Muscle depletion can predict the survival rate of cancer patients. In 2014, the Asian Working Group for Sarcopenia published a consensus on sarcopenia, recommending screening steps and diagnostic thresholds for sarcopenia.^[32]^ Asian Working Group for Sarcopenia also recommended the dynamic changes in muscle mass, strength, physical performance, weakness, instrumental activities of daily living, and basic activities of daily living over a certain period of time as outcome indicators of sarcopenia, and suggested that fear of falling and urinary incontinence be included as outcome indicators for sarcopenia research.

The paper ranked second in citations (3519 citations) are the consensus on sarcopenia definition and diagnosis revised by the European Working Group on Sarcopenia in Older People working group during the period 2018 to 2022. The organization updated the consensus in 2018, proposing the cutoff point for female Appendicular Skeletal Muscle Mass/height^2^ to be <6.0 kg/m^2^. In 2019, the working group revised the cutoff point for female Appendicular Skeletal Muscle Mass/height^2^ to be <5.5 kg/m^2^.^[33]^

### 4.4. Theme development analysis

The strategic coordinate map is based on the common word matrix and clustering analysis of keywords, and visual graphics are used to display the results of data analysis. The research method is based on Law’s et al^[34]^ suggestion in 1988 that “strategic coordinates” can be used to describe the links between the topics within the research field to reflect the research status of each topic and to describe the links between the internal keywords of each topic to reflect the degree of development of the topic. The strategic coordinate map drawn by SciMAT can visually present the development status of each research topic during this period, and the transfer of research topics in different periods on the strategic coordinate map reflects the evolution state of the topic. The nodes in the coordinate map represent the corresponding clustering topics, and the numbers are the number of relevant documents on the topic, and the number of documents reflects the degree of attention to the topic. The abscissa is Centrality, which indicates the degree of correlation between the topic and other topics in the field. The larger the value, the closer the connection between the topic and the topics in the field, and it belongs to the core topic in the research field. The ordinate is Density, which indicates the strength of the connection between the keywords within the topic. The larger the value, the closer the connection within the topic and the higher the maturity of the topic development.

The strategic coordinate map is divided into 4 quadrants (Q). Q1 is the upper-right Q, containing central topics. Q2 is the upper-left Q, containing highly developed and isolated topics. Q3 is the lower-left Q, containing emerging or declining topics, and Q4 is the lower-right Q, containing basic and horizontal topics. The strategic coordinate maps, related bibliometric indicators, and evolution of research topics for the 5 research periods in this study are as follows.

1. Topics of the 1993 to 2002 research period (n = 3): *AGE, MOTOR UNIT*, and *MEN*

**Figure 4.**
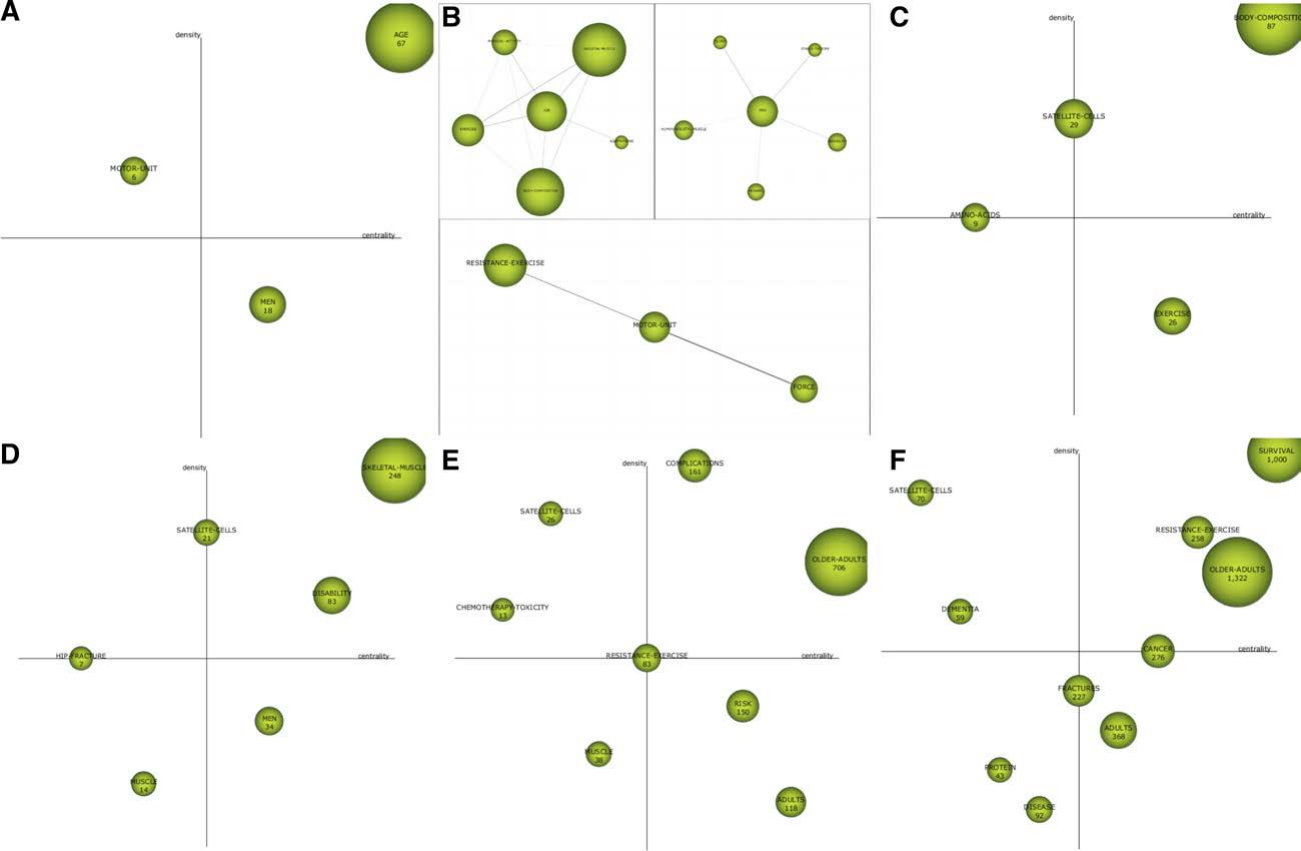
Thematic strategy map for each research period. (A) The thematic strategy map for the period 1993–2002 (based on the number of documents). (B) Clustering network of topics in the research period 1993–2002. (C) Thematic strategy map for the period 2003–2007 (based on the number of documents). (D) Thematic strategy map for the period 2008–2012 (based on the number of documents). (E) Thematic strategy map for the period 2013–2017 (based on the number of documents). (F) Thematic strategy map for the period 2018–2022 (based on the number of documents).

**Figure 5.**
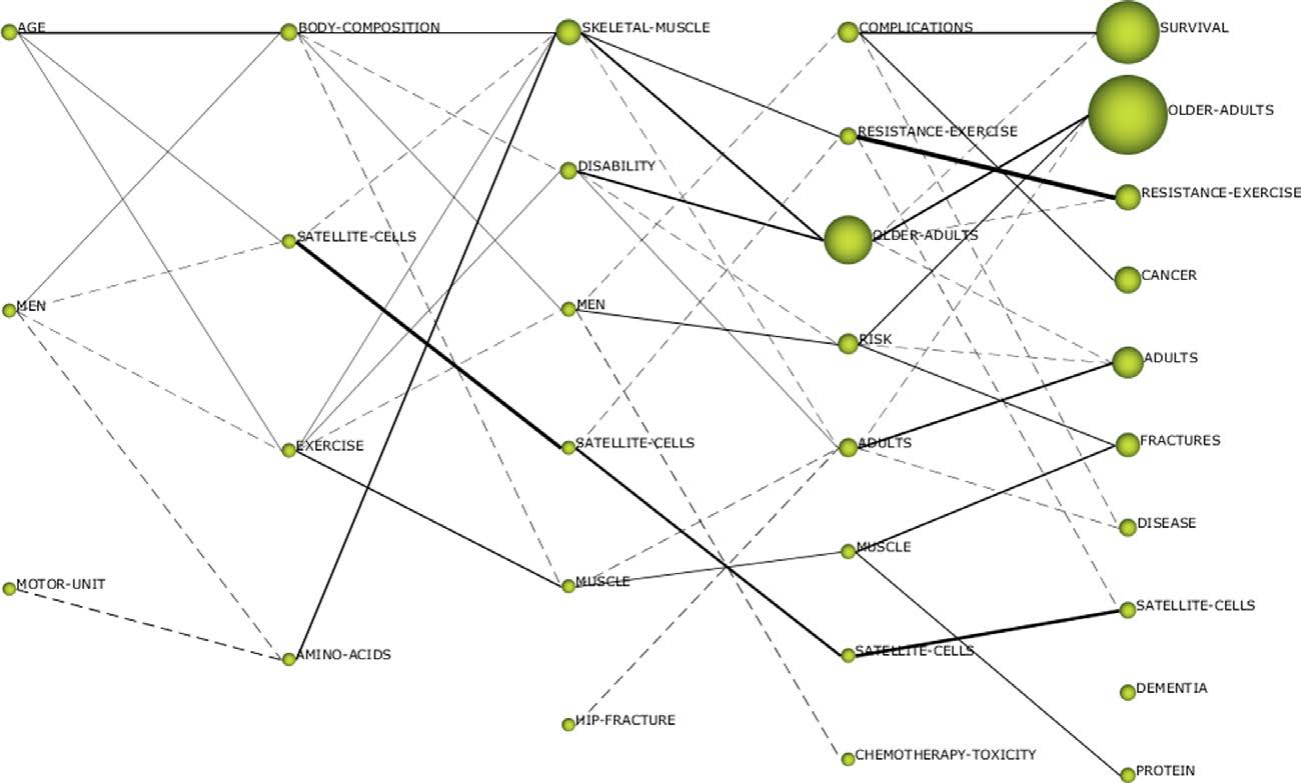
Evolutionary pathway of sarcopenia themes during the study period 1993–2022 (from left to right, they are the research period from 1993 to 2002, 2003 to 2007, 2008 to 2012, 2013 to 2017, and 2018 to 2022).

Table 2 and Figure 4A and B shows the strategic map during the research period from 1993 to 2002. The theme of Q1 is *AGE*, which has a high centrality and density value, indicating that it has received a lot of attention, is relatively developed, has a greater impact on later research, and is a hot topic for subsequent research. As shown in Figure 5, there are many keywords related to *AGE*, the most closely related of which are *EXERCISE, PHYSICAL ACTIVITY, SKELETON MUSCLE*, and *ADAPTATIONS*. The theme of Q2 is *MOTOR UNIT*, which is a theme with high maturity and low centripetal nature. The centrality value is 6.01 and the density value is 11.2. The correlation within the theme is strong, and the correlation with other topics is weak. The keywords closely related to *MOTOR UNIT* are *RESISTANCE EXERCISE* and *FORCE*. The theme of Q4 is *MEN*, with a center value of 11.9 and a density value of 5.09, which is closely related to the keywords *DISABILITY, HUMANS, HUMAN SKELETON MUSCLE, OLDER*, and *STABLE ISOTOPE*.

**Table 2:** Evaluation indicators of various topics during the research period from 1993 to 2002.

Theme	Centrality	Density	Number of documents	Number of citations
*AGE*	19.07	12.39	67	13,524
*MOTOR UNIT*	6.01	11.2	6	1317
*MEN*	11.9	5.09	18	4641

1. Topics of the 2003 to 2007 research period (n = 4): *BODY COMPOSITION, SATELLITE CELLS, EXERCISE*, and *AMINO ACIDS*

Topic *BODY COMPOSITION* is located in the Q1 area and has a high centrality and density value (Table 3, Fig. 4C). The clustering network (see Figure S1, Supplemental Digital Content, http://links.lww.com/MD/J390) shows that the topic is interrelated with multiple keywords, of which the most closely related keywords are *PHYSICAL ACTIVITY* and *SKELETAL MUSCLE. SATELLITE CELLS* are on the density axis, with a center value and a density value of 5.58 and 7.07, respectively. The clustering network shows that this topic is most closely related to *HUMAN SKELETAL MUSCLE. AMINO ACIDS* is on the central axis, and its central value and density value are 2.67 and 3.75, respectively. The keywords are closely related to it are *STABLE ISOTOPE* and *RESISTANCE-EXERCISE.* The theme of Q4 is *EXERCISE*, and the centerness and density values are 9.15 and 2.56, respectively. The clustering network shows that the keyword most closely related to it is *HORMON*.

**Table 3:** Evaluation indicators of various topics during the research period from 2003 to 2007.

Theme	Centrality	Density	Number of documents	Number of citations
*BODY COMPOSITION*	13.89	8.79	87	12,120
*SATELLITE CELLS*	5.58	7.07	29	3787
*EXERCISE*	9.15	2.56	26	2760
*AMINO ACIDS*	2.67	3.75	9	1094

1. Topics of the 2008 to 2012 research period (n = 6): *SKELETAL MUSCLE, DISABILITY, MEN, SATELLITE CELLS, MUSCLE*, and *HIP FRACTURE*

As shown in Table 4 and Figure 4D, the topics of Q1 are *SKELETAL MUSCLE* and *DISABILITY*. They are the topics with the highest centrality and development maturity during this period. The centrality is 16.17 and 6.68, respectively, while the density values are 7.09 and 4.12, respectively. The *SKELETAL MUSCLE* of this research period is closely related to several keywords, such as *BODY COMPOSITION, EXERCISE*, etc. (see Figure S2, Supplemental Digital Content, http://links.lww.com/MD/J391). *SATELLITE CELLS* is located on the density axis and are closely related to the keywords *FIBER TYPE, MYOBLASTS*, etc. *HIP FRACTURE* is located on the central axis, with a centerness value of 0.63 and a density value of 2.37. The clustering network display is connected to the keyword *RISK FACTORS*.

**Table 4:** Evaluation indicators of various topics during the research period from 2008 to 2012.

Theme	Centrality	Density	Number of documents	Number of citations
*SKELETAL MUSCLE*	16.17	7.09	248	24,227
*DISABILITY*	6.68	4.12	83	6595
*MEN*	5.62	1.62	34	2665
*SATELLITE CELLS*	2.83	4.56	21	1132
*MUSCLE*	2.38	0.94	14	1505
*HIP FRACTURE*	0.63	2.37	7	275

The theme of Q3 is *MUSCLE*, which is in a position with low maturity and centripetal nature, with center and density values of 2.38 and 0.94, respectively. The clustering network shows that the keywords related to *MUSCLE* are *PHYSICAL PERFORMANCE* and *RAT*. The theme of Q4 is *MEN*, which is in a position with a high degree of center but immature development. It is a theme with a certain potential for development. The center is 5.62 and the density value is 1.62. The clustering network shows that the relationship between the internal keywords of the MEN topic is very weak, and the relatively strong ones are *DETERMINANT, FAT*, and *RISK*.

1. Topics of the 2013 to 2017 research period (n = 8): *COMPLICATIONS, RESISTANCE EXERCISE, OLDER ADULTS, RISK, ADULTS, MUSCLE, SATELLITE CELLS*, and *CHEMOTHERAPY TOXICITY*

The topics of Q1 are *OLDER ADULTS* and *COMPLICATIONS*, which are in a position of high maturity and centripetal, and are the core research topics of this research period. Their centerness values are 23.33 and 101.67, respectively, while the density values are 7.96 and 24.69, respectively (Table 5, Fig. 4E). The themes of Q2 are *SATELLITE CELLS* and *CHEMOTHERAPY TOXICITY*, which are in the position of high maturity and low centripetal degree. The centerness values are 9.57 and 0.93, respectively, and the density values are 0.95 and 6.94, respectively. The theme of Q3 is *MUSCLE*, which is in a position where both maturity and centripetal degree are low. The clustering network (see Figure S3, Supplemental Digital Content, http://links.lww.com/MD/J392) shows that the internal connection of the theme IS loose. The topics of Q4 are *RISK* and *ADULTS*, which are highly centralized and low-maturity, and are the core topics in the field with great potential for development.

**Table 5:** Evaluation indicators of various topics during the research period from 2013 to 2017.

Theme	Centrality	Density	Number of documents	Number of citations
*COMPLICATIONS*	101.67	24.69	2645	128,904
*RESISTANCE EXERCISE*	45.16	4.74	594	33,595
*OLDER ADULTS*	23.33	7.96	390	25,845
*RISK*	37.47	3.87	445	23,226
*ADULTS*	13.69	4.35	113	7631
*MUSCLE*	9.53	6.82	141	7840
*SATELLITE CELLS*	9.57	0.95	42	3234
*CHEMOTHERAPY TOXICITY*	0.93	6.94	32	1616

1. Topics of the 2018 to 2022 research period (n = 10): *SURVIVAL, OLDER ADULTS, RESISTANCE EXERCISE, CANCER, ADULTS, FRACTURES, DISEASE, SATELLITE CELLS, DEMENTIA*, and *PROTEIN*

The theme of Q1 is that *SURVIVAL, OLDER ADULTS,* and *RESISTANCE EXERCISE* are in a position of high maturity and centripetal degree, with high research popularity and influence, and mature development. It is the central theme in the field. The centerness is 13.26, 12.6, and 6.95, respectively, and the density values are 7.57, 4.53, and 5.14, respectively. Among them, the volume of posts on *RESISTANCE EXERCISE* topics has increased significantly, and the development has become more mature (Table 6, Fig. 4F).

**Table 6:** Evaluation indicators of various topics during the research period from 2018 to 2022.

Theme	Centrality	Density	Number of documents	Number of citations
*SURVIVAL*	13.26	7.57	1000	11,965
*OLDER ADULTS*	12.6	4.53	1322	16,994
*RESISTANCE EXERCISE*	6.95	5.14	258	4924
*CANCER*	6.91	2.81	276	3351
*ADULTS*	6.22	1.01	368	4163
*FRACTURES*	3.33	2.8	227	2846
*DISEASE*	3.2	0.31	92	848
*SATELLITE CELLS*	0.52	5.18	70	854
*DEMENTIA*	0.61	2.93	59	664
*PROTEIN*	1.45	0.51	43	555

The topics of Q2 are *SATELLITE CELLS* and *DEMENTIA*, with centerness values of 0.52 and 0.61, respectively, and density values of 5.18 and 2.93. The clustering network (see Figure S4, Supplemental Digital Content, http://links.lww.com/MD/J393) shows that the keywords closely related to *DEMENTIA* are *COGNITIVE IMPAIRMENT, DECLINE*, and *IMPAIRMENT*. The topics of Q3 are *PROTEIN* and *DISEASE*, which are in a position of low maturity and low centripetal degree. The internal links of the topics are loose, and they are on the edge of research. They are newborn topics. Their centerness values are 3.83, 3.28, and 3.49, and their density values are 1.62, 0.93, and 0.69, respectively. The theme of Q4 is *ADULTS*, which is in a position with low maturity and high centripetal degree, with a centrality value of 6.22 and a density value of 1.01.*FRACTURES* is on the density axis and is a subject with high maturity and moderate centripetal degree. The centerness value is 3.33 and the density value is 2.8.*CANCER* is on the axis of centrality, with moderate maturity and high centripetal degree, with a centrality value of 6.91 and a density value of 2.81.

It can be seen from the strategic diagrams of each stage that the content of research in the field of sarcopenia is getting richer and richer, and over time it has gradually deepened into mechanism research and multi-system integration research. *RESISTANCE EXERCISE* is evolving to a position of high maturity and centripetal degree, suggesting that it has always been a hot topic in the field of sarcopenia. The *SATELLITE CELLS* theme has gradually evolved from a position with moderate maturity and the centripetal degree to a position with high maturity and low centripetal degree, suggesting that the research on myosatellite cells in the field of sarcopenia has become more mature.

### 4.5. Thematic dynamic graph evolution analysis

The dynamic evolution diagram of the topic (Fig. 5) displays the evolution trends of the major topics in the 5 research time zones over time in the form of a data stream, to facilitate the analysis and tracking of the dynamic development of topics in the field of sarcopenia in a continuous period. In the evolution chart, each vertical column represents a period, from left to right, namely 1993–2002, 2003–2007, 2008–2012, 2013–2017, and 2018–2022. The nodes in the figure represent research topics, and the size of the nodes is proportional to the number of research topics, the connection between the nodes represents the flow direction of data. The solid line indicates that there is a common main keyword between the 2 topics, which represents the mainstream evolution direction, and the dotted line indicates that there is a common secondary keyword between the node topics, which represents the mainstream evolution direction. The depth and thickness of the connection color are proportional to the degree of correlation between the 2 themes. The dark and thick connection colors indicate that the 2 themes have a high degree of similarity, strong correlation, and strong evolutionary ability; nodes that are not connected indicate that the theme appeared separately during the time period and did not form a connection with the theme of the previous and subsequent periods.

With the help of the dynamic evolution map of the topic, the development and changes of research topics in the field of sarcopenia in the entire research process are analyzed, and then important topic evolution paths are identified in the evolution trend. From a horizontal perspective, there were few research topics from 1993 to 2002, and the degree of attention was low, but the degree of correlation with the later period was high. In 2003–2007 and 2008–2012, the number of research topics increased, new topics appeared, and the degree of correlation between topics increased, and each topic was able to evolve and develop through mainstream or branch directions. The number of research topics continued to increase from 2013 to 2017 and from 2018 to 2022. New topics emerged significantly, and the research’s popularity increased. The mainstream direction and the branch direction were able to evolve and develop. Some topics died out, and the evolution path between topics became more complex. The stable development of topics in the mainstream evolution direction has continued to become a hot topic in the field of research, and the number of new topics and stable topics has increased, but it has not become a research center.

The *AGE* group evolved into 3 groups *BODY COMPOSITION, SATELLITE CELLS,* and *EXERCISE* during the research period from 2003 to 2007, indicating that the research on this topic has been further refined. The *SATELLITE CELLS* group was the second-ranked group in the research period from 2003 to 2007, but it ranked fourth, seventh, and eighth in the subsequent research period, indicating that the research on this topic has gone through a period of overheating, mature development but gradually withdrew from the hot topic of sarcopenia research. The *MEN* group evolved into *BODY COMPOSITION, SATELLITE CELLS, EXERCISE,* and *AMINO ACIDS* in the research period from 2003 to 2007. After 2 research periods, it evolved into the second-ranked *OLDER ADULTS* and the sixth-ranked *FRACTURES* group in the research period from 2018 to 2022, indicating that the topic has a strong evolutionary ability. The MOTOR UNIT theme evolved into the *AMINO ACIDS* theme during the research period from 2003 to 2007, the *SKELETAL MUSCLE* theme evolved into the number 1 group during the research period from 2008 to 2012, the second and third groups during the research period from 2013 to 2017, and the second and third groups during the research period from 2018 to 2022, indicating that the topic has a strong evolutionary ability and is the core topic in the research field in recent years with great potential for development.

Overall, the number of topics and the number of published articles have been increasing over time. When a certain research content accumulates to a certain extent, new branches will arise. New topics continue to emerge at all stages, and the research content in the field of sarcopenia continues to be enriched. Each group has continuously evolved into new groups over time, and the groups at various stages of time are closely related. Except for *HIP FRACTURE* and *DEMENTIA*, there are no isolated groups, and there are intersections between the various research groups. The research in the field of sarcopenia is in a stage of rapid development, and the maturity is not high. The research topics have changed significantly in various periods. The topics have undergone differentiation, fusion, metastasis, and regeneration. The phenomenon is obvious, and the evolution process is stable.

## 5. Conclusion

Research in the field of sarcopenia has a history of only 30 years. This article used SciMAT software to divide the relevant literature on sarcopenia research into 5 time zones. In the early stage of research, there were fewer publications and research topics, but the development of research topics was mature. In the later stage, the number of research topics increased continuously, and new topics emerged, showing a diversified development trend. The dynamic evolution paths of research topics in each period are distinct and clear, and the evolution process is stable, with potential development topics becoming core topics in the field. Mature and stable professional topics in the later period, such as “RESISTANCE EXERCISE” and “SURVIVAL,” belong to the core topics, while emerging topics such as “FRACTURES” and “PROTEIN” belong to marginal topics, indicating that the research on muscle-bone metabolism in the sarcopenia field still needs to be further explored. In addition, the “CANCER” topic is a research topic with great development potential.

## Author contributions

**Conceptualization:** Juan Chen.

**Data curation:** Juan Chen, De-jun Wang, Yi-chen Zhang.

**Methodology:** De-jun Wang.

**Project administration:** Yan-hong SU.

**Supervision:** Yan-hong SU.

**Validation:** Yi-chen Zhang.

**Visualization:** De-jun Wang, Yi-chen Zhang.

**Writing – original draft:** Juan Chen.

**Writing – review & editing:** Juan Chen, De-jun Wang, Yan-hong SU.

## Supplementary Material








