# Supplementary figures and images for: Evolutionary overview of sarcopenia – bibliometric study on the Web of science: A review
Source: Medicine (Baltimore). 2023 Jul 28;102(30):e34500. doi: 10.1097/MD.0000000000034500 (PMC10378895; doi:10.1097/MD.0000000000034500)

Supplementary Figure 1 Clustering network of themes for the study period 2003-2007.

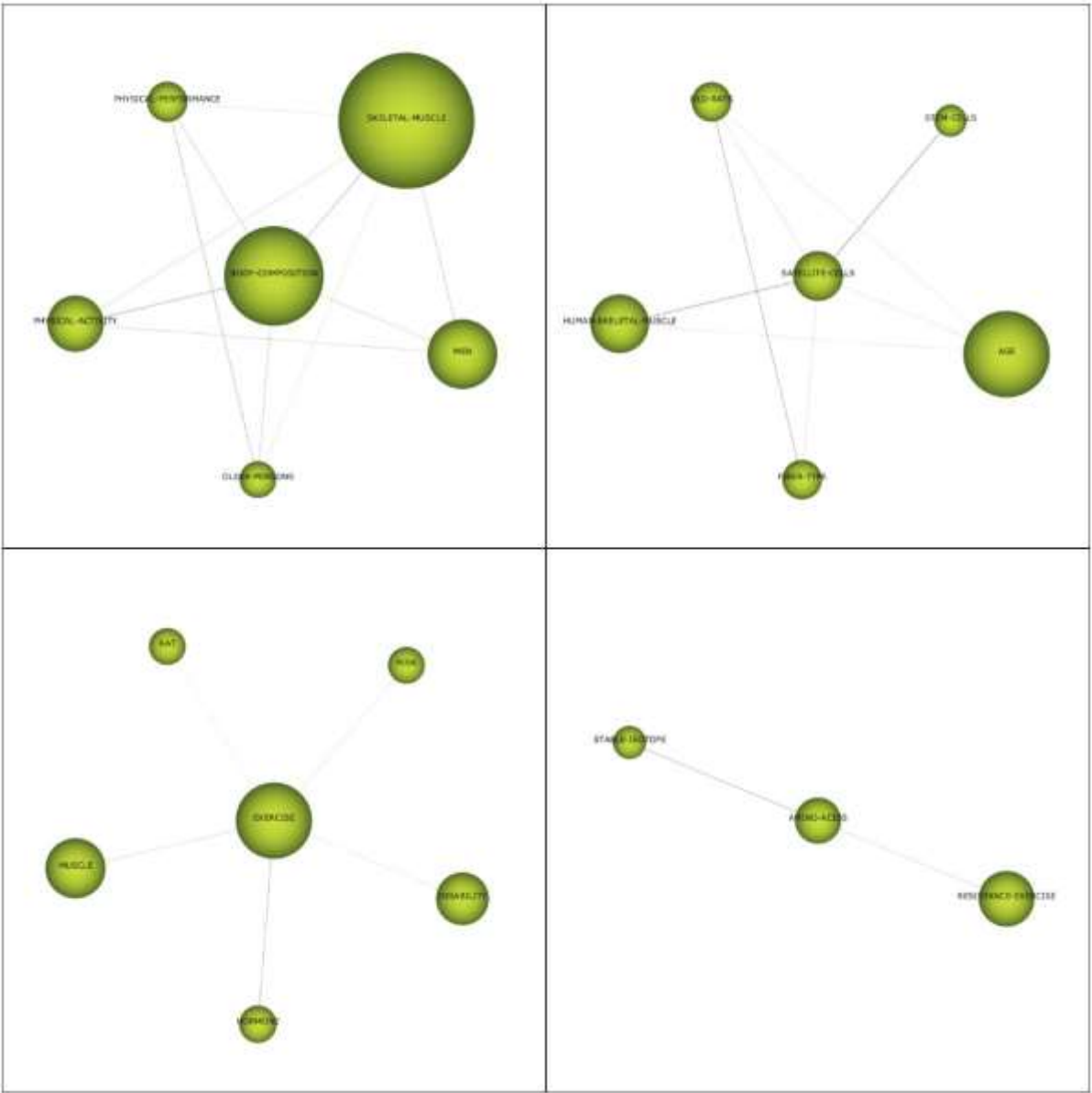

Supplement: Supplementary file 1 [file medi-102-e34500-s001.pdf]

**Supplementary Figure 2 Clustering network of themes for the study period 2008-2012.**

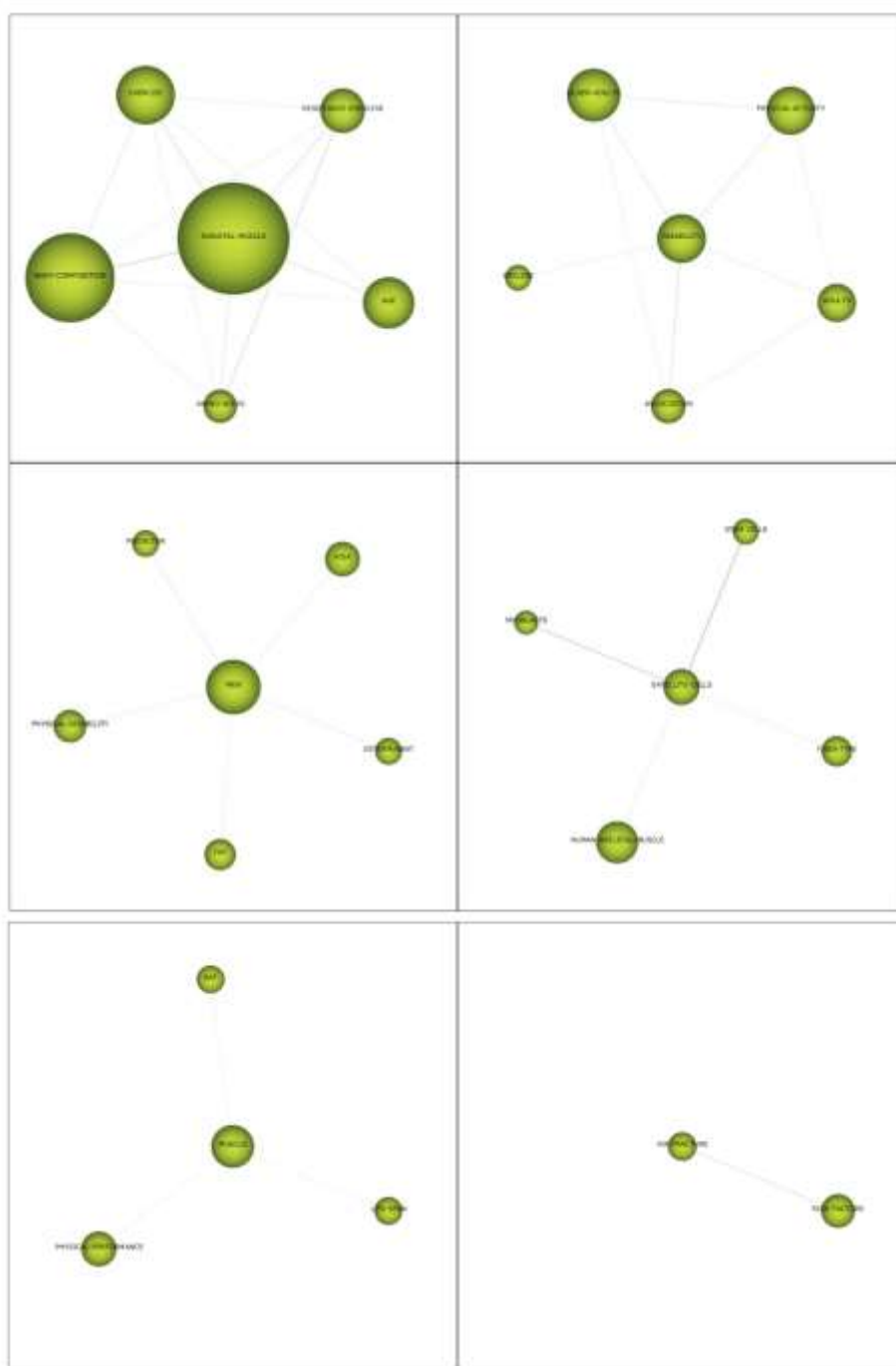

Supplement: Supplementary file 2 [file medi-102-e34500-s002.pdf]

**Supplementary Figure 3 Clustering network of themes for the study period 2013-2017.**

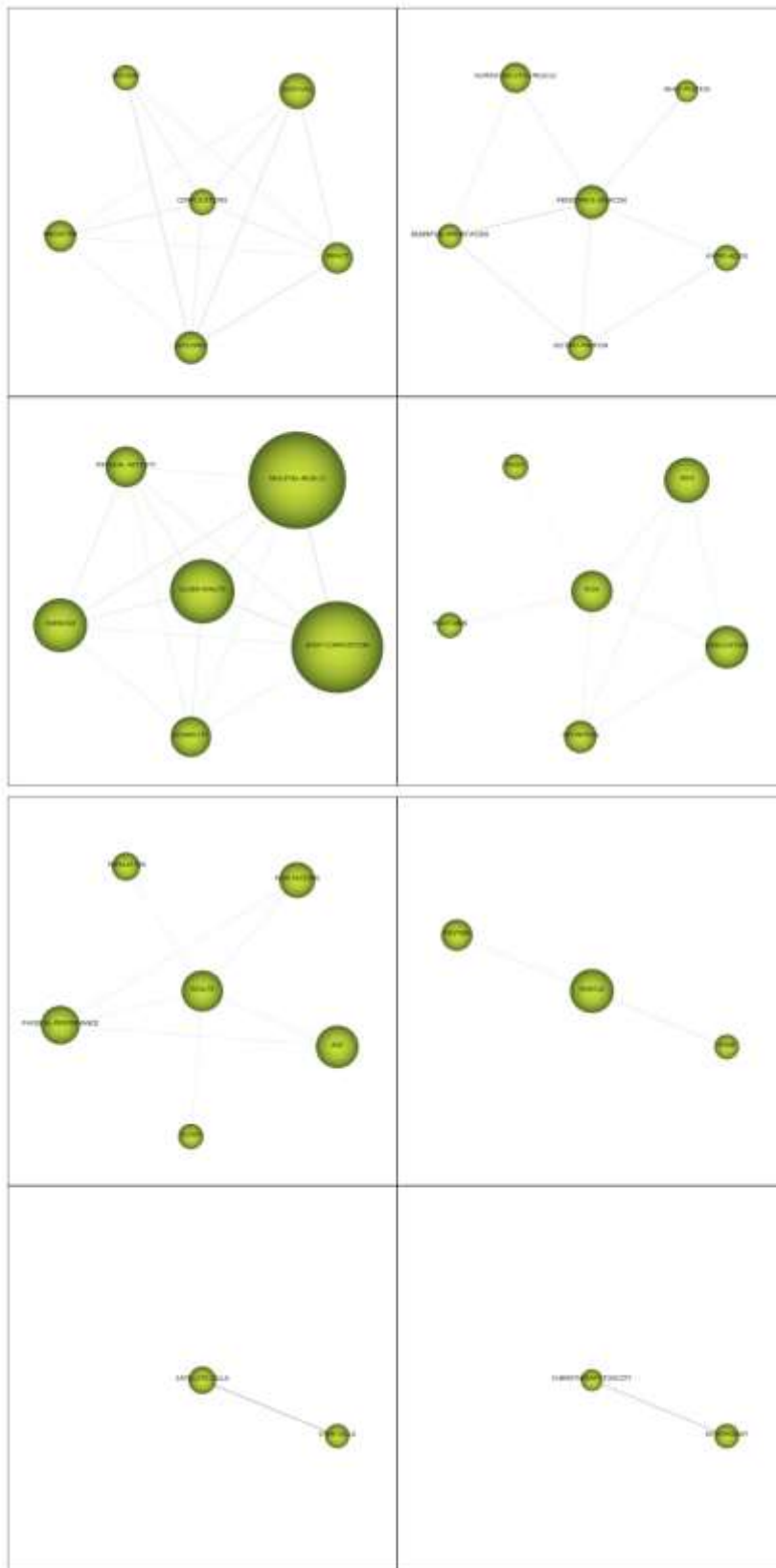

Supplement: Supplementary file 3 [file medi-102-e34500-s003.pdf]

Supplementary Figure 4 Clustering network of themes for the study period 2018-2022.

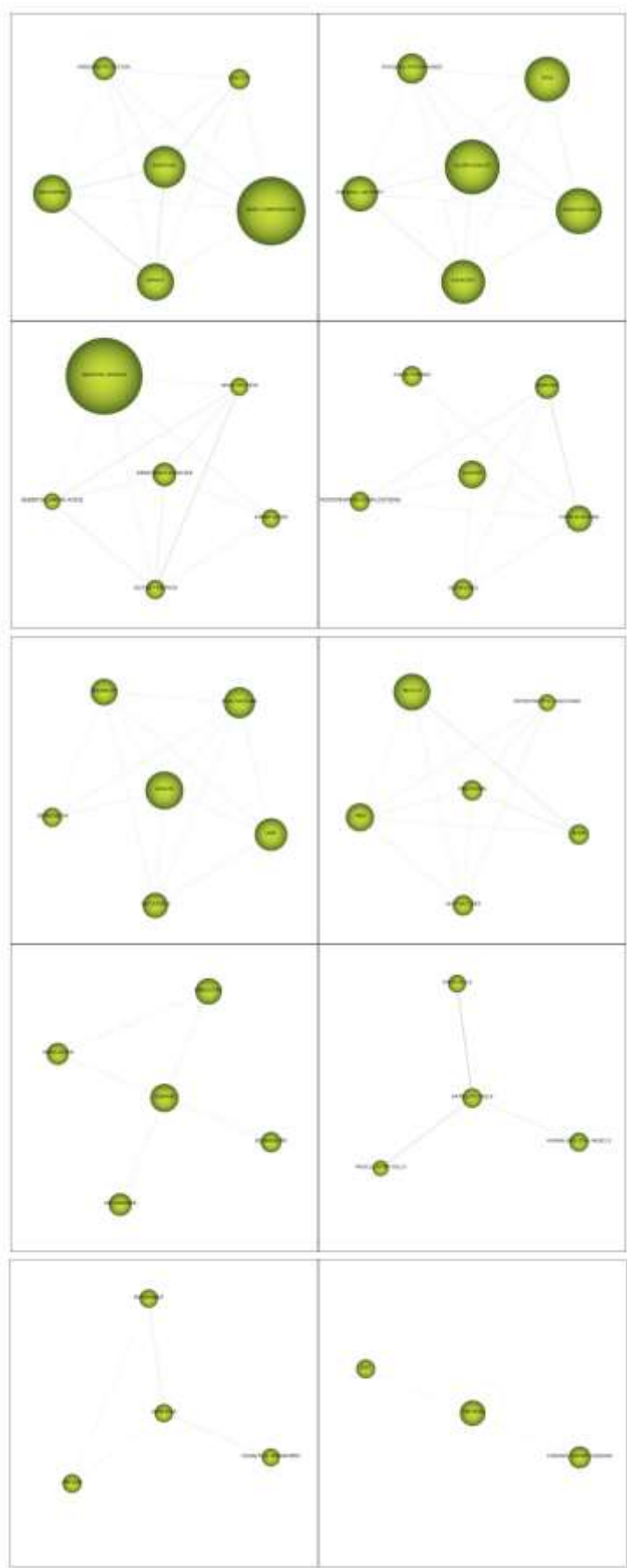

Supplement: Supplementary file 4 [file medi-102-e34500-s004.pdf]
